# Metagenomic Next-Generation Sequencing for the Diagnosis of Infectious Uveitis: A Comprehensive Systematic Review

**DOI:** 10.3390/v17060757

**Published:** 2025-05-26

**Authors:** Isabele Pardo, Luciana P. S. Finamor, Pedro S. Marra, Julia Messina G. Ferreira, Maria Celidonio Gutfreund, Mariana Kim Hsieh, Yimeng Li, João Renato Rebello Pinho, Luiz Vicente Rizzo, Takaaki Kobayashi, Daniel J. Diekema, Michael B. Edmond, Paulo J. M. Bispo, Alexandre R. Marra

**Affiliations:** 1 Faculdade Israelita de Ciências da Saúde Albert Einstein, Hospital Israelita Albert Einstein, São Paulo 05652-900, SP, Brazil; maria.cgutfreund@einstein.edu.br (M.C.G.); joao.pinho@einstein.br (J.R.R.P.); alexandre-rodriguesmarra@uiowa.edu (A.R.M.); 2 Department of Ophthalmology, Universidade Federal de São Paulo, São Paulo 05508-090, SP, Brazil; luciana.finamor@grupofleury.com.br; 3 University of California San Francisco, San Francisco, CA 94143, USA; pedro.marra@ucsf.edu (P.S.M.); yimeng.li@ucsf.edu (Y.L.); 4 Hospital Israelita Albert Einstein, Instituto Israelita de Ensino e Pesquisa Albert Einstein, São Paulo 05652-900, SP, Brazil; julia.ferreira@einstein.br (J.M.G.F.); lvrizzo@einstein.br (L.V.R.); 5 The Program of Hospital Epidemiology, University of Iowa Health Care, Iowa City, IA 52242, USA; mariana-kimhsieh@uiowa.edu; 6 LIM03/07, Hospital das Clínicas da Faculdade de Medicina da Universidade de São Paulo, São Paulo 05403-000, SP, Brazil; 7 Department of Internal Medicine, Carver College of Medicine, University of Iowa, Iowa City, IA 52242, USA; takaaki-kobayashi@uiowa.edu (T.K.); daniel-diekema@uiowa.edu (D.J.D.); 8 Division of Infectious Diseases, Department of Internal Medicine, College of Medicine, University of Kentucky, Lexington, KY 40536-0298, USA; 9 Department of Medicine, School of Medicine, West Virginia University, Morgantown, WV 26506-9111, USA; michael.edmond1@wvumedicine.org; 10 Department of Ophthalmology, Harvard Medical School, Infectious Disease Institute, Massachusetts Eye and Ear, Boston, MA 02114, USA; paulo_bispo@meei.harvard.edu

**Keywords:** systematic literature review, metagenomic NGS, infectious uveitis, diagnostic methods

## Abstract

**Background**: Infectious uveitis is a potentially sight-threatening condition that requires timely and accurate pathogen identification to guide effective therapy. However, conventional microbiological tests (CMTs) often lack sensitivity and the inclusiveness of pathogen detection. Metagenomic next-generation sequencing (mNGS) offers an unbiased approach to detecting a broad range of pathogens. This review evaluates its diagnostic performance in detecting infectious uveitis. **Methods**: A systematic search across multiple databases identified studies assessing the use of mNGS for diagnosing infectious uveitis. The included studies compared mNGS to CMTs, including polymerase chain reaction (PCR), culture, serology, and the IGRA (Interferon-Gamma Release Assay). The study characteristics; the detection rates; and the sensitivity, specificity, and predictive values were extracted. The sensitivity and specificity of mNGS were calculated using CMTs as a reference. **Results**: Twelve studies comprising 859 patients were included. The sensitivity of mNGS compared to that of CMTs ranged from 38.4% to 100%, while specificity varied between 15.8% and 100%. The commonly detected pathogens included varicella-zoster virus, cytomegalovirus, *Toxoplasma gondii*, and herpes simplex virus. In some cases, mNGS outperformed PCR in viral detection, aiding diagnosis when the standard methods failed. However, contamination risks and inconsistent diagnostic thresholds were noted. **Conclusions**: mNGS enables the diagnosis of infectious uveitis, particularly for viral causes, but its variable performance and standardization challenges warrant further investigation.

## 1. Introduction

Infectious uveitis is a serious intraocular inflammatory condition that can cause severe vision impairment and blindness if not promptly identified and treated. This condition is often difficult to diagnose because it can be caused by various pathogens, including viruses, bacteria, fungi, and parasites [1]. These infections usually exhibit overlapping clinical features, which can mimic non-infectious uveitis [1]. In this scenario, one of the primary challenges in diagnosing infectious uveitis is accurately identifying the causative pathogen to guide appropriate treatment. However, the small sample size of the intraocular specimens often limits the sensitivity and specificity of the traditional diagnostic methods, such as culture and intraocular antibody detection, which are also time-consuming [1,2,3]. Moreover, the eye’s immune privilege and localized immunological mechanisms can further hinder pathogen detection, contributing to diagnostic challenges [4].

Metagenomic next-generation sequencing (mNGS) is a novel technique that has the potential to improve pathogen detection in infectious uveitis [5]. It allows for the comprehensive identification of a broad spectrum of pathogens, including those difficult to culture or identify using the traditional methods [5,6]. Its ability to generate vast sequencing data for unbiased pathogen detection has led to its increasing global adoption as a diagnostic tool [7,8]. However, mNGS also presents challenges, such as a poor specificity, which can lead to false positives due to contamination and the detection of clinically nonsignificant organisms. Additionally, interpreting mNGS data requires advanced bioinformatics tools [3,5,9,10]. Here, we performed a comprehensive systemic review to assess the clinical performance of mNGS compared to that of the conventional microbiological tests (CMTs) in diagnosing infectious uveitis and to identify the barriers that currently hinder the broad applicability of mNGS as a diagnostic tool for infectious uveitis.

## 2. Materials and Methods

### 2.1. Systematic Literature Review and Inclusion and Exclusion Criteria

This review followed the Preferred Reporting Items for Systematic Reviews and Meta-Analysis (PRISMA) statement [11]. Institutional Review Board approval was not required. This study was registered on Prospero (https://www.crd.york.ac.uk/PROSPERO/) on 7 December 2024 (registration number CRD42024618873). In this study, we defined infectious uveitis as inflammation involving all three anatomical regions of the uveal tract—anterior, intermediate, and posterior segments—resulting from infections by various pathogens, including bacteria, viruses, fungi, and parasites. The complete search strategy is demonstrated in Supplementary S1 [12]. We defined metagenomic next-generation sequencing (mNGS) as a laboratory method that uses shotgun next-generation sequencing to detect any genome present in a clinical sample [5]. The typical mNGS workflow involves three key phases. First, during sample preparation, ocular fluids (aqueous or vitreous humor) undergo DNA/RNA extraction using commercial kits, followed by the generation of cDNA libraries (for RNA-seq) and sequencing library preparation using fragmentation for short-read sequencing (not performed for long-read sequencing) and adapter ligation. Because of the small size of intraocular specimens, most methods employed for the sequencing of intraocular fluids rely on the use of PCR to amplify and concentrate the sequencing libraries. Second, libraries are quantified and pooled, and sequencing is performed on platforms such as Illumina (short-read and high-accuracy) or Oxford Nanopore (long-read and real-time), generating from hundreds of thousands to millions (1–20 million) of reads of per samples depending on the method/platform used and the number of samples pooled in one run. Third, bioinformatic analysis filters human sequences and aligns the remaining reads to pathogen databases [9,13,14]. On the other hand, the conventional microbiological tests (CMTs) included antigenic tests, culture, the enzyme-linked immunosorbent assay (ELISA) for the detection of local antibody production in aqueous humor, PCR (polymerase chain reaction), and the IGRA (Interferon-Gamma Release Assay). We defined a positive result in the CMT group if at least one of the diagnostic methods mentioned was positive in a single sample.

This review included manuscripts published up to 17 February 2025. There were no language restrictions. The inclusion criteria for studies were original research manuscripts; published in peer-reviewed, scientific journals; case series studies; observational study designs; and involved individuals that had infectious uveitis and were submitted to mNGS-based pathogen detection.

Commentaries, case reports, studies on pediatric populations, studies with overlapping samples of patients, and non-peer-reviewed studies were excluded. Studies in which there was no comparison between mNGS and a CMT were also excluded.

### 2.2. Search Strategy

We performed literature searches in PubMed, Embase (Elsevier Platform), the Cochrane Central Register of Controlled Trials, Web of Science, the Cumulative Index to Nursing and Allied Health (CINAHL), and Scopus. The complete search strategy is demonstrated in Supplementary S1. Also, we assessed the reference lists of included articles to find papers not identified from the initial literature search.

**Figure 1:**
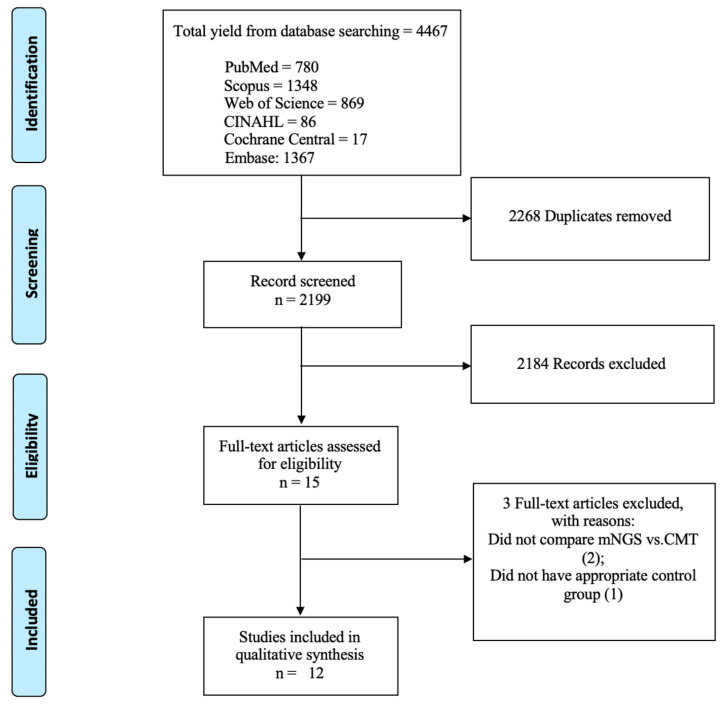
Flow diagram of literature search adapted from PRISMA flow chart.

This study employed the PICO framework [15]. We focused on patients with infectious uveitis (P) to compare metagenomic next-generation sequencing (I) against conventional microbiological tests (C). Our primary outcomes (O) were diagnostic performance, clinical impact, and possible barriers to the broad implementation of mNGS for routine clinical use. To filter the 4467 studies acquired from the databases, the titles and/or abstracts were evaluated by two investigators (I.P. and A.R.M.). Studies with overlapping patients, incomplete data, or that did not meet the PICO inclusion criteria were excluded using Rayyan app through consensus [16]. After the first filtration, 15 studies were fully read, and 12 were included in this systematic literature review (Figure 1).

### 2.3. Data Abstraction and Quality Assessment

Of six independent reviewers (I.P., P.S.M., M.C.G., J.M.G.F., M.K.H., and Y.L.), two independently extracted the data for each article using a standardized abstraction form (Supplementary S2). The reviewers resolved disagreements by unanimity. The standardized abstraction form included a record of the data on study design, publication year and calendar time, demographic characteristics of the selected population, mNGS methodology, analyzed CMTs, sample types, detection rates and accuracy comparison between methods, commonly identified pathogens, mNGS advantages, limitations, clinical impact, and conclusions.

We assessed the risk of bias and methodological quality of the included studies using a modified Downs and Black scale [17]. All the original questions from this scale were applied, except for question #27, which was adapted to a yes/no response, resulting in a maximum achievable score of 28 points. Two reviewers independently scored each study, resolving disagreements through consensus. The scores were categorized as follows: good quality (>18 points), fair quality (15–18 points), and poor quality (<15 points).

## 3. Results

### 3.1. Characteristics of Included Studies in This Systematic Literature Review

Twelve studies [18,19,20,21,22,23,24,25,26,27,28,29] were included in this systematic review (Figure 1 and Table 1). Five studies were retrospective cohort studies [21,22,25,27,29], three were prospective cohort studies [24,26,28], three were case series [18,19,20], and one was a transversal study [23]. Most studies were performed in China (six studies) [18,25,26,27,28,29], followed by the USA (three studies) [20,21,22], the Netherlands (one study) [19], Japan (one study) [23], and South Korea (one study) [24]. These studies were conducted between 2010 and 2023, varying from 5 to 70 months in duration [18,19,20,21,22,23,24,25,26,27,28,29].

In qualitative analysis, twelve studies including 859 patients, of whom 525 (61.1%) had infectious uveitis, evaluated the diagnostic value of mNGS. Of 459 infectious uveitis cases with a reported etiology, 206 (44.9%) were viral [20,21,23,24,25,26,27,28,29]. The majority of the studies used the Illumina platform to perform mNGS (eight studies) [19,20,21,22,25,26,27,29], followed by Oxford Nanopore Technology sequencers (two studies) [23,24], the Beijing Genomics Institute (one study) [28], and MGI Sequencers (one study) [18]. Of the twelve studies evaluated, nine focused on DNA testing [18,19,21,23,24,25,26,27,29], while three examined RNA testing [20,22,28]. The vast majority of the studies sampled both aqueous and vitreous humors (seven studies) [18,20,22,24,25,26,27], although four studies solely focused on sampling aqueous humor [19,23,28,29], and one study on sampling vitreous humor [21]. There was a vast range of sample volume used for mNGS testing (20–100 µL) [18,20,21,22,23,24,26,27,28,29], with a mode of 50 µL [21,24,28,29], which was similar to the amounts used for each CMT sample.

Eleven studies compared the positivity rates of mNGS and a CMT, defined as the proportion of samples yielding positive results indicative of infection [18,19,20,21,22,24,25,27,28,29]. In three studies, mNGS yielded a higher positivity rate than the CMT [18,21,22]; in three studies, the positivity rates were equivalent [19,20,24]; and in five studies, the positivity rates were higher with the CMT [23,25,27,28,29]. Overall, the positivity rates ranged from 9.7% to 100% for mNGS and from 19.2% to 100% for the CMT [18,19,20,21,22,23,24,25,27,28,29]. One study did not separately report rates for each method, providing only comparative sensitivity and specificity values [25].

### 3.2. Sensitivity and Specificity of mNGS Using Conventional Microbiological Tests (CMTs) as Reference

Using CMTs as the reference, the sensitivity of mNGS ranged from as low as 38.4% [29] to as high as 100%, depending on the comparator and the study design [18,19,20]. Specificity also varied widely, ranging from 15.8% [26] to 100% [19]. When using culture-based methods as the reference, mNGS generally demonstrated more sensitivity, with studies such as Doan 2016 and Lee 2023 reporting 100% sensitivity [20,24]. In contrast, when targeted PCR was used as the comparator, mNGS sensitivity was more variable, ranging from 40% to 100% across multiple studies [18,19,21,22,23,24,25,26,27,28,29]. Additionally, one study specifically evaluated the diagnostic performance of mNGS for **viral infections** in intraocular fluid samples, reporting good sensitivity (90.7%), specificity (100%), a positive predictive value (100%), and a negative predictive value (81.0%) [27]. Positive predictive values (PPVs) and negative predictive values (NPVs) were less consistently reported, but also varied considerably, reflecting differences in the patient populations, pathogen load, and the laboratory procedures.

### 3.3. Sensitivity and Specificity of mNGS and CMTs Using Clinical Diagnosis as Reference

Two studies quantitatively compared mNGS and the clinical diagnosis [18,26]. Sensitivity ranged from 87.8% to 96.9%, while specificity varied from 58.4% to 69.2% [18,26]. Two studies compared CMTs and the clinical diagnosis. Sensitivity ranged from 59.4% to 75.0%, while specificity varied from 96.2% to 100% [18,20].

### 3.4. Pathogens Identified

The most commonly detected pathogens using mNGS included varicella-zoster virus (VZV), cytomegalovirus (CMV), *Toxoplasma gondii*, herpes simplex virus (HSV-1 and HSV-2), Epstein–Barr virus (EBV), and *Klebsiella pneumoniae*. Some studies, such as Doan 2016 and Doan 2021, reported the unique identification of *Cryptococcus neoformans* and *Pithomyces chartarum*, respectively, which were not detected using the conventional methods [20,22]. Notably, Qian 2023 highlighted the difficulty of mNGS in detecting Mycobacterium tuberculosis, indicating that protocol optimization is necessary for intracellular bacterial and fungal pathogens [25]. Doan 2016 highlighted how mNGS aided in the diagnosis of a bilateral chronic rubella uveitis in a patient without a previous known etiology [20].

One study evaluated the difference in the detection rates of vitreous humor and aqueous humor using mNGS. The vitreous humor samples showed a slightly increased pathogen detection rate (85.7–100.0%) when compared to that of aqueous humor using mNGS (80.0–88.9%) in most types of infection, except for viral infection, with 80.0% and 86.4% rates, respectively. However, statistically, both the samples are suitable for mNGS testing [26].

Two studies evaluated the diagnostic value of mNGS in identifying coinfections in uveitis [27,29]. Sun et al. reported two cases of coinfection, one involving VZV and CMV, and another involving HSV-2 and *Treponema pallidum*. When comparing mNGS and PCR, mNGS successfully identified both the pathogens in each coinfection. Although the PCR panel test correctly detected both VZV and CMV in the first case, it failed to identify *Treponema pallidum* in the second coinfection, detecting only HSV-2 [27]. Yu et al. reported one case with both HSV-1 and Epstein–Barr virus, where mNGS was positive for both pathogens, while the ELISA assay of aqueous humor only detected HSV-1. On the other hand, one study reported a false positive call from mNGS (*Klebsiella pneumoniae*) for a potential VZV-*Klebsiella* coinfection in a patient with only chronic VZV, correctly confirmed by the ELISA [29].

Regarding methodological quality assessed by the modified Downs and Black scale, two studies were classified as good [24,26], six as fair [21,23,25,27,28,29], and four as poor [18,19,20,22].

## 4. Discussion

mNGS has emerged as a valuable diagnostic tool for identifying pathogens in cases of infectious uveitis. In this systematic literature review, mNGS demonstrated wide ranges of sensitivity (38–100%) and specificity (15.8–100%) when compared to those of the CMTs as the reference standard. Notably, when using clinical diagnosis as the reference, mNGS showed more sensitivity (87.8–96.9%), but less specificity (58.4–69.2%), whereas CMTs had less sensitivity (59.4–75.0%), but more specificity (96.2–100%).

mNGS showed equal or superior positivity rates compared to those of the CMT in six out of eleven studies that reported this value (54.5%), supporting its potential role as a complementary or alternative diagnostic approach for infectious uveitis. The particularly high detection rates for viruses such as CMV, VZV, HSV-1, and HSV-2 reinforce the diagnostic utility of mNGS, especially for viral etiologies of infectious uveitis. However, the detection rate of mNGS among the infected patients ranged from 9.7% to 100%, while the CMTs showed a detection rate range from 19.2% to 100%. The majority of included studies (eight out of twelve; 66.7%) were classified as having good or fair methodological quality, strengthening our confidence in these results.

### 4.1. Sensitivity and Specificity of mNGS in Viral Detection Using CMTs as Reference

mNGS demonstrated more sensitivity in detecting viral pathogens using CMTs as reference. Studies by Doan et al. (2016, 2017, 2021), Sun 2024, and Qian 2023 reported sensitivity rates above 80% for viral detection [20,21,22,25,27]. For instance, Sun 2024 observed a sensitivity of 90.7% for mNGS in viral infectious uveitis, supporting the reliability of mNGS in identifying intraocular viral pathogens [27].

### 4.2. Advantages over Conventional Methods

A significant advantage of mNGS is its unbiased, hypothesis-free approach, which enables the detection of a wide array of pathogens without prior assumptions. Unlike PCR, which requires specific primers, mNGS can identify both known and novel viruses (Table 2). This was evident in Doan 2017, where mNGS uncovered 6 additional pathogens either not detected or not tested with pathogen-directed PCRs in 8 samples (22%) [21]. Also, Doan 2021 showed that mNGS identified pathogens not considered in the differential diagnosis in 9.7% of cases [21,22]. Beyond common viruses, mNGS identified rare or unexpected agents, such as rubella virus and human T-cell leukemia virus (HTLV-1). In Doan et al., 2016 study, mNGS was able to detect rubella virus in a case of chronic bilateral uveitis, suggesting the presence of long-term viral replication in the eye [20]. This highlights the potential of metagenomic to uncover latent viral infections that may not be detected by routine diagnostic tests. However, it is important to note that the ability to detect some of these viruses depends on whether RNA sequencing is included in the mNGS workflow, as DNA sequencing alone would not capture the RNA viruses detected in the studies.

In terms of diagnostic performance, mNGS has also demonstrated superiority in sensitivity when compared to CMTs using clinical diagnosis as the reference standard. Notably, mNGS showed more sensitivity (87.8–96.9%), but less specificity (58.4–69.2%) [15,23] whereas CMTs exhibited less sensitivity (56.4–75.0%), but more specificity (96.2–100%) [15,17]. This trade-off underscores mNGS strength in identifying a broader spectrum of pathogens, including those potentially missed by the CMTs (Table 2), though it may also yield more false positives due to its heightened sensitivity.

Moreover, mNGS can simultaneously detect atypical infections, a capability that is especially valuable in immunocompromised patients who are at higher risk for viral coinfections. Consistent with the findings from our systematic review, Nguyen et al. reported a case in which mNGS was highly valuable for detecting intraocular Monkeypox virus in an immunocompromised patient with chronic lymphocytic leukemia who presented without classic prodromal symptoms or skin lesions [30]. Also, Xu et al. and Hu et al. demonstrated that the metagenomic sequencing of vitreous fluid diagnosed acute retinal necrosis associated with pseudorabies virus infection. As this disease has rapid progression and can cause severe visual impairment, mNGS’s fast and unbiased diagnostic capability can be crucial [31,32]. The capacity of mNGS to simultaneously detect atypical pathogens is therefore particularly relevant in immunocompromised populations. In contrast, within this review, mNGS showed limited and variable sensitivity in detecting *M. tuberculosis*, emphasizing the need for methodological improvements in ocular tuberculosis diagnosis. This challenge is further supported by the minimal sensitivity observed with multiplex PCR for detecting *M. tuberculosis* DNA in intraocular samples from clinically suspected cases, likely due to the paucibacillary nature of this disease [33].

Furthermore, mNGS provides genomic insights beyond pathogen identification. In one study included in our review, Koyanagi et al. demonstrated that mNGS can infer drug resistance genes, supporting targeted therapy decisions [23]. External studies not included in this review also highlight its potential. Gu et al. showed how metagenomics helped detect gene variations, such as loss-of-function variations in the RNR gene in VZV, that might lead to impaired virulence with an increased duration of the plateau phase during treatment [34]. Doan et al. (2017) also reported that mNGS could infer the phenotypic behavior of identified pathogens [21]. Three out of seven CMV samples analyzed in this study had mutations in the gene UL97 (phosphotransferase), which confers ganciclovir and valganciclovir resistance [21]. Such capability enhances patient management by enabling precision medicine approaches by predicting drug resistance profiles based on the genomic data.

### 4.3. Challenges and Limitations

Despite its promising applications, mNGS faces several limitations. One primary concern is the risk of false positive results due to contamination from the laboratory environment, kit reagents, or the periocular and ocular surface microbiota microbes that can be introduced during sample collection. Multiple studies, including those by Cai 2024, Koyanagi 2023, Qian 2024, and Sun 2024, highlighted difficulties in differentiating true pathogens from contaminants [18,23,26,27]. Qian 2024 found that while mNGS exhibited 82.4% sensitivity, its specificity was only 15.8% [26], suggesting a tendency to over-detect pathogens. The identification of non-pathogenic or commensal microbes can complicate clinical interpretation, necessitating robust bioinformatics filtering and threshold settings. Variations in sequencing platforms, such as Illumina and Oxford Nanopore, which have substantial difference in the total sequencing outputs, could also contribute to discrepancies in sensitivity and specificity.

Additionally, the high cost, labor-intensive workflow, and complex bioinformatics analyses required for mNGS implementation were identified as barriers to its widespread clinical adoption [22,23]. The turnaround times, although improving, remain longer than those of PCR, making mNGS less feasible for urgent clinical decision making.

Also, while the testing of vitreous humor demonstrated slightly higher pathogen detection rates, particularly for non-viral infections [26], testing aqueous humor samples may represent a more feasible and less invasive alternative for mNGS-based diagnostics in routine clinical practice. The vast range of sample volumes used in mNGS testing (20–100 µL) [18,20,21,22,23,24,26,27,28,29], a quantity similar for each CMT sample, shows an area of possible improvement for mNGS, as this method could potentially streamline the diagnosis of infectious uveitis by supporting the use of a single and comprehensive test to allow for broad pathogen detection. As ocular samples are limited, the reduction in the amount of fluid required for mNGS detection can become an essential advantage over CMT.

### 4.4. Implications for Future Research and Clinical Practice

This study has several limitations. All the studies included in this analysis were non-randomized, which may introduce potential bias in our findings. A lack of randomization increases the risk of confounding variables, preventing us from establishing causal relationships. Additionally, the limited number of studies that reported data comparing mNGS to clinical diagnosis prevented us from conducting meta-analysis with the pooled sensitivity and specificity values [18,26]. Furthermore, most studies did not provide sufficient independent information regarding the mNGS performance differences according to the anatomical classification of uveitis. Lastly, it is important to acknowledge that the variability in sample types (aqueous vs. vitreous humor), sequencing platforms (e.g., Illumina and Oxford Nanopore), and sequencing depth across the included studies may have influenced the reported sensitivity and specificity. This underscores the need for methodological standardization to ensure a more consistent diagnostic performance. Consequently, this study highlights the need for further research to expand the scope of mNGS assessment of the diagnosis of infectious uveitis.

To maximize the clinical utility of mNGS in diagnosing infectious uveitis, several advancements are needed. First, the standardization of diagnostic thresholds and bioinformatics pipelines is essential to improve specificity and reduce false positives. Second, future research should focus on optimizing the sequencing depth and pathogen detection sensitivity, particularly for low-viral-load infections. Lastly, cost reduction strategies, such as targeted mNGS panels, may enhance feasibility for routine clinical practice. Future studies should address these issues and clarify specific clinical scenarios where mNGS could be preferred over the traditional methods, such as in immunocompromised patients, atypical cases, or when conventional diagnostics are inconclusive. Additionally, there is a lack of studies directly comparing the mNGS diagnostic performance against clinical diagnosis as the reference standard. This gap limits our ability to fully assess mNGS clinical diagnostic accuracy, especially regarding its sensitivity and specificity in clinically confirmed infectious uveitis cases. Across the studies, the nucleic acid-based tests (mNGS and PCR) demonstrated more agreement and a better performance in the early or acute phases of infection. In contrast, the antibody-based tests showed prolonged detection windows, especially in chronic cases. This disparity highlights the importance of choosing diagnostic tools based on the disease stage. Addressing this limitation should be a priority in future research to better define the diagnostic utility of mNGS in routine ophthalmologic practice.

## 5. Conclusions

In conclusion, this systematic review highlights the clinical value of mNGS in diagnosing infectious uveitis, particularly for viral etiologies and in cases where the traditional diagnostic methods have failed. mNGS has demonstrated the ability to identify unexpected pathogens and viral coinfections, offering a hypothesis-free diagnostic approach. While some studies reported sensitivity levels approaching 100%, this suggests that mNGS may achieve a diagnostic performance comparable to that of PCR in certain contexts. However, given that CMTs served as the reference standard in most included studies, the true comparative accuracy of mNGS versus PCR remains difficult to establish. Nevertheless, several barriers, including specificity concerns, contamination control, costs, and the need for methodological standardization, particularly in bioinformatics analyses, still need to be addressed to enhance its broader clinical applicability.

## Figures and Tables

**Table 1:** Summary of characteristics of studies included in this systematic literature review.

First Author, Year, Location, Study Design, Study Period in # of Months and [Dates]	Participants (n) and Characteristics	mNGS Type/Sequencer/Sample Type, and Conventional Microbiological Tests (CMT)	Detection Rate of mNGS and Conventional Microbiological Tests Among Infected Patients		Performance Characteristics Using Various Conventional Microbiological Tests or Clinical Diagnosis as the Reference as Indicated	Most Common Detected Pathogens	Advantages/ Clinical Impact of mNGS	Limitations of mNGS	Conclusions	D and B Score (Max = 28)
			mNGS	CMT						
Cai, 2024 China Prospective Case Series Study NR	58 cases of intraocular infections (32 cases of infectious uveitis and endogenous/exogenous endophthalmitis and 26 cases of non infectious uveitis). Demographic characteristics were not reported	DNA testing using MGI sequencer. Sampling of aqueous and vitreous humor. Compared with PCR, culture, antibody, purified protein derivative (PPD) test, T-spot test, and chest computed tomography	96.9% (31/32)	59.4% (19/32)	mNGS vs. PCR: Sensitivity: 100% Specificity: 50%	VZV, CMV, *Toxoplasma gondii*, EBV, *Klebsiella pneumoniae*, *Mycobacterium tuberculosis*	Diagnostic performance of mNGS compared to clinical diagnosis: Sensitivity: 96.9% Specificity: 69.2%. CMT compared to clinical diagnosis, on the other hand, demonstrated: Sensitivity: 59.4% Specificity: 96.2%	Sampling and the lab environment may be a source of significant interference in mNGS	mNGS showed more sensitivity, but less specificity. It increased the detection rate of infectious uveitis pathogens, but might result in false positives	13
de Groot-Mijnes 2017, Netherlands Retrospective Case Series NR	11 patients with possible infectious uveitis (6 positive for infectious uveitis). Demographic characteristics were not reported	DNA testing using Illumina. Sampling of aqueous humor. Compared with PCR	100% (6/6)	PCR: 100% (6/6)	mNGS vs. PCR: Sensitivity: 100% Specificity: 100% PPV: 100%	*Toxoplasma gondii* and VZV	NR	Non-human reads constitute only less than 1% of the total number of reads. Moreover, ocular fluid sampling inherently introduces floral bacteria in the sample	mNGS can be applied for the diagnosis of infectious uveitis	8
Doan, 2016 USA Case series NR	6 samples of patients with uveitis (4 cases of infectious uveitis and 2 cases on non-infectious uveitis). Demographic characteristics were not reported	RNA testing using Illumina. Sampling of aqueous and vitreous humor. Compared with PCR and culture	100% (4/4)	PCR: 50% (2/4) Culture: 100% (1/1)	mNGS vs. CMT: Sensitivity: 100% Specificity: 66.6% NPV: 75% PPV: 100%	*Cryptococcus neoformans*, *Toxoplasma gondii*, rubella, and HSV-1	One case of chronic intraocular rubella virus infection was detected first through mNGS (positive impact in 16.6% of cases). CMT compared to clinical diagnosis, on the other hand, demonstrated: Sensitivity: 75% Specificity: 100%	Difficulty to discriminate between microbes that are present as a result of contamination and those that are actually causing disease.	mNGS can identify fungi, parasites, and DNA and RNA viruses in minute volumes of intraocular fluid samples	11
Doan, 2017 USA Retrospective Cohort Study 60 months [2010–2015]	67 samples of patients with presumed uveitis (37 positive for infectious uveitis). Demographic characteristics were not reported	DNA testing using Illumina. Sampling of vitreous humor. Compared with PCR	89.2% (33/37)	PCR: 83.8% (31/37)	mNGS vs. PCR: Sensitivity: 87.1% Specificity: 77.8% PPV: 77.1% NPV: 87.5%	*Klebsiella pneumoniae*, *Candida dubliniensis*, HSV-2, HTLV-1, HHV-6, CMV	mNGS can apply sequence information to infer the phenotypic behavior of the identified pathogen. Eight samples (22%) tested by mNGS resulted in 6 additional pathogens either not detected or not tested with pathogen-directed PCRs	DNA-sequencing alone cannot detect RNA viruses (e.g., rubella).	Metagenomic DNA sequencing is highly concordant with pathogen-directed PCRs.	15
Doan 2021, USA Retrospective cohort study 19 months [June 2018–December 2019]	41 samples of patients with presumed ocular infection (16 positive for infectious uveitis). Demographic characteristics were not reported	RNA testing using Illumina. Sampling of aqueous and vitreous humor. Compared with PCR	100% (16/16)	PCR: 75% (12/16)	mNGS vs. PCR: Sensitivity: 100% Specificity: 92.6% PPV: 87.5% NPV: 100%	HSV-1, HSV-2, CMV, VZV, *Toxoplasma gondii*, rubella	mNGS identified pathogens not on the differential diagnosis for 9.7% (4/41) of the samples. Two pathogens were solely identified with it	The costs, the labor-intensive library sequencing workflow and the bioinformatics required for metagenomics are the major barriers	mNGS can identify known and unknown pathogens from intraocular fluid samples of patients with presumed intraocular infections.	12
Koyanagi 2023, Japan Transversal study 70 months [April 2017–January 2023]	45 patients with suspected uveitis (22 positive for infectious uveitis). The mean age was 53.8 years and 42.2% of participants were female	DNA testing using the Oxford Nanopore MinION sequencers. Sampling of aqueous humor. Compared with PCR	59% (13/22)	100% (22/22)	mNGS vs. PCR: Sensitivity: 59%	HSV-1, HSV-2 VZV, CMV, EBV	Comprehensive search for pathogen and drug resistance	Nanopore metagenome analysis results contain considerable noise, and that contamination control is necessary	Nanopore metagenomic results contained considerable noise and were less sensitive compared to conventional tests	15
Lee, 2023, South Korea Prospective cohort study 5 months [September 2020–January 2021]	8 patients with intraocular infectious, 5 diagnosed with endogenous endophthalmitis/uveitis and 3 having exogenous endophthalmitis. The mean age was 54.4 years and 37.5% of participants were female	DNA testing using the Oxford Nanopore MinION sequencer. Sampling of aqueous or vitreous humor. Compared with culture and PCR	100% (5/5)	Culture:66.6% (2/3) PCR: 100% (2/2)	mNGS vs. Culture: Sensitivity: 100% mNGS vs. PCR: Sensitivity: 100%	*Klebsiella pneumoniae*, *Clostridium septicum*, and CMV	Nanopore performed better and had an average sample-to-answer time lower than traditional pathogen diagnostic methods	Nanopore sequencing has previously had a more limiting role because of higher error rates. Improvements to the method have led to its increased prevalence.	Nanopore mNGS is a promising diagnostic tool that can rapidly and accurately identify the causative pathogen in infectious uveitis	19
Qian, 2023 China Retrospective Cohort Study 22 months [May 2019–February 2021]	14 patients with ocular symptoms (11 cases of intraocular infection and 3 cases on non-infectious uveitis). The mean age was 37 years old and 53.3% were female	DNA testing using Illumina. Sampling of aqueous and vitreous humor. Compared with PCR, culture and T-SPOT.	72.7% (8/11)	Culture: 100% (5/5) PCR: 100% (3/3) TSPOT: 100% (3/3)	mNGS vs. Culture: Sensitivity: 100% mNGS vs. PCR: Sensitivity: 100% mNGS vs. TSPOT Sensitivity: 33.3%	HSV-1, VZV, EBV, *Staphylococcus aureus*, *Klebsiella pneumoniae*, *Mycobacterium tuberculosis* and *Aspergillus flavus*	NR	mNGS showed difficulty in detecting *Mycobacterium tuberculosis*. The mNGS protocols should be optimized for the detection of intracellular bacterial and fungal pathogens	NGS could be helpful in determining pathogens in cases of suspected intraocular infection	17
Qian, 2024 China Prospective Cohort Study 32 months [January 2019–August 2021]	488 patients with suspected intraocular infections, including cases of exogenous and endogenous endophthalmitis plus infectious uveitis (288 positive infectious). The mean age was 47.3 years and 39.5% of participants were female	DNA testing using Illumina. Sampling of aqueous and vitreous humor. Compared with PCR, culture and detection antibody	NR	NR	mNGS vs. CMT: Sensitivity: 82.4% Specificity: 15.8%	*Staphylococcus epidermidis*, *Candida albicans*, EBV, *Treponema pallidum*, and *Toxoplasma gondii*	Diagnostic performance of mNGS compared to clinical diagnosis for viral uveitis: Sensitivity: 87.8% Specificity: 58.4%	mNGS detects more contaminate microbes than other methods, producing more false positive results	mNGS helps in a rapid, independent, and impartial diagnosis of bacterial and other intraocular infections	22
Sun 2024, China, Retrospective cohort study 38 months [May 2020–August 2023]	70 patients with suspected viral infectious uveitis (53 cases of infectious uveitis and 17 cases on non-infectious uveitis). The mean age was 45.3 years and 48.5% of participants were female	DNA testing using Illumina platform. Sampling of aqueous and vitreous humor. Compared with PCR and serology	90.6% (NR)	PCR: 91.7% (11/12)	mNGS vs. PCR: Sensitivity: 90.9%	VZV, HSV-1, HSV-2, CMV, *Bartonella henselae*, *Toxoplasma gondii*, and *Treponema pallidum*	mNGS was more valuable in detecting rare pathogens than PCR. Overall viral sensitivity: 90.7%, specificity: 100%, PPV: 100%, and PNV: 81.0%	False positive results due to data pollution. No commonsense on diagnostic threshold, that is, what value of DNA reads was the low limit for a positive result	mNGS is a sensitive and valuable method to detect virus in intraocular fluid samples	16
Wang, 2022, China Prospective cohort study 10 months [March 2017–December 2017]	31 patients with infectious uveitis. The mean age was 42.5 years and 48.4% of participants were female	RNA testing using BGI (Beijing Genomics Institute). Sampling of aqueous humor. Compared with qPCR and enzyme-linked immunosorbent assay (ELISA) combined with Witmer-Desmonts coefficient (WDC) evaluation	9.7% (3/31)	PCR: 19.2% (5/26) ELISA: 64.5% (20/31)	mNGS vs. PCR: Sensitivity: 40% Specificity: 95.5% PPV: 66.7% NPV: 87.5% mNGS vs. ELISA: Sensitivity: 15% Specificity: 100% PPV: 100% NPV: 39.3%	CMV, VZV, rubella	NR	The sensitivity from aqueous humor by mNGS was not satisfactory, which could be associated with the low pathogen titers in the small volume of samples and relatively low reading	mNGS is a potential etiologic diagnosis tool to seek different intraocular viral pathogens, although its sensitivity still needs to be improved	18
Yu, 2024 China Retrospective Cohort Study 10 months [May 2020–February 2021]	20 patients with infectious uveitis. The mean age was 38.35 years and 50% of participants were female	DNA testing using Illumina platform. Sampling of aqueous humor. Compared with PCR and Elisa	35% (7/20)	ELISA: 100% (13/13) PCR: 60% (3/5)	mNGS vs. CMTs: Sensitivity: 38.4% Specificity: 71.4%	CMV, VZV, HSV-1, HSV-2, EBV, *Pseudomonas aeruginosa*, *Bacillus megaterium*, *Klebsiella pneumoniae*, *Toxocara*	Using a small volume of sample, mNGS enables a comprehensive and unbiased analysis, allowing the identification of pathogens with detailed taxonomic resolution	Sensitivity and specificity of mNGS can be influenced by several critical factors, including sequencing depth, pathogen load, proportion of host-derived background, and potential contamination during the assay	mNGS identifies a broad range of pathogens, including viruses, bacteria, fungi, and previously unrecognized agents in infectious uveitis	16

Abbreviations: CMV, cytomegalovirus; D and B, Downs and Black; EBV, Epstein–Barr virus; HSV, herpes simplex virus; HTLV, human T-cell leukemia virus; NPV, negative predictive value; NR, not reported; PCR, polymerase chain reaction; PPV, positive predictive value; VZV, varicella-zoster virus.

**Table 2:** Comparative diagnostic performance of mNGS vs. CMTs.

Method	Strengths	Limitations	Best Clinical Use
mNGS	Untargeted detection; novel pathogen identification	Less specificity; cost/bioinformatics	Atypical/ immunocompromised cases
PCR	More specificity; rapid (hours)	Targeted. Limited inclusiveness and some misses coinfections	Suspected viral/ toxoplasmic uveitis
Culture	Gold standard for viability	Less sensitivity; slow (days to weeks)	Fungal/non-viral infections
Serology	Detects chronic infections	Cannot confirm active infection	Syphilis/tuberculosis

Abbreviations: mNGS, metagenomic next-generation sequencing; CMTs, conventional microbiological tests.

## Data Availability

The data are available in the References and the Supplemental Material.

## Supplementary Materials

The following supporting information can be downloaded at: https://www.mdpi.com/article/10.3390/v17060757/s1, File: Supplemental Material mNGS, Supplementary S1: Search terms and strategy; Supplementary S2: Standardized Data Abstract Form.
