# Supplementary material for: Metagenomic Next-Generation Sequencing for the Diagnosis of Infectious Uveitis: A Comprehensive Systematic Review
Source: Viruses. 2025 May 26;17(6):757. doi: 10.3390/v17060757 (PMC12197381; doi:10.3390/v17060757)
Supplement: Supplementary file 1 [file viruses-17-00757-s001.zip › viruses-3624453-supplementary.pdf]

Metagenomic Next-Generation Sequencing for the Diagnosis of Infectious Uveitis: A Comprehensive Systematic Review

Supplemental Material

Isabele Pardo [1,*], Luciana P. S. Finamor [2], Pedro S. Marra [3], Julia Messina G. Ferreira [4], Maria Celidonio Gutfreund [1], Mariana Kim Hsieh [5], Yimeng Li [3], João Renato Rebello Pinho [1,6], Luiz Vicente Rizzo [4], Takaaki Kobayashi [7,8], Daniel J. Diekema [7], Michael B. Edmond [9], Paulo J. M. Bispo [10] and Alexandre R. Marra [1,7]

1 Faculdade Israelita de Ciências da Saúde Albert Einstein, Hospital Israelita Albert Einstein,
São Paulo 05652-900, SP, Brazil; maria.cgutfreund@einstein.edu.br (M.C.G.); joao.pinho@einstein.br (J.R.R.P.); alexandre-rodriguesmarra@uiowa.edu (A.R.M.)

2 Department of Ophthalmology, Universidade Federal de São Paulo, São Paulo 05508-090, SP, Brazil; luciana.finamor@grupofleury.com.br

3 University of California San Francisco, San Francisco, CA 94143, USA; pedro.marra@ucsf.edu (P.S.M.); yimeng.li@ucsf.edu (Y.L.)

4 Hospital Israelita Albert Einstein, Instituto Israelita de Ensino e Pesquisa Albert Einstein,
São Paulo 05652-900, SP, Brazil; julia.ferreira@einstein.br (J.M.G.F.); lvrizzo@einstein.br (L.V.R.)

5 The Program of Hospital Epidemiology, University of Iowa Health Care, Iowa City, IA 52242, USA;
mariana-kimhsieh@uiowa.edu

6 LIM03/07, Hospital das Clínicas da Faculdade de Medicina da Universidade de São Paulo,
São Paulo 05403-000, SP, Brazil;

7 Department of Internal Medicine, Carver College of Medicine, University of Iowa,
Iowa City, IA 52242, USA;
takaaki-kobayashi@uiowa.edu (T.K.); daniel-diekema@uiowa.edu (D.J.D.)

8 Division of Infectious Diseases, Department of Internal Medicine, College of Medicine, University of Kentucky, Lexington, KY 40536-0298, USA

9 Department of Medicine, School of Medicine, West Virginia University, Morgantown, WV 26506-9111, USA;
michael.edmond1@wvumedicine.org

10 Department of Ophthalmology, Harvard Medical School, Infectious Disease Institute, Massachusetts Eye and Ear, Boston, MA 02114, USA;
paulo_bispo@meei.harvard.edu

* Correspondence: isabele.pardo@einstein.edu.br; Tel.: +55-(11)-99441-1100

1. Contents

1. **Cover Page** ……………….……………………..................................1

2. **Contents** ……………………….………...2

3. **Supplemental S1**. Search terms and strategy …………….…………..3

4. **Supplemental S2:** Standardized Data Abstract Form …………………..7

Supplemental S1. Search terms and strategy

mNGS	Infectious Uveitis	NOT - Genetic diseases
**MESH**	**MESH**	**MESH**

High Throughput Nucleotide Sequencing	Uveitis	Genetic Diseases, X-Linked
Metagenomics	Panuveitis	Genetic Diseases, Y-Linked
molecular diagnostic techniques	Iritis	Genetic Diseases, Inborn
	Chorioretinitis	
	Retinitis	
	Eye infections	
	Pars Planitis	
	Iridocyclitis	
	Panophthalmitis	
	Choroiditis	
FREE TERMS	FREE TERMS	FREE TERMS
Next Generation sequencing	Infectious Uveitis	inherited diseases
mNGS	Diffuse uveitis	genetic diseases
NGS	Anterior uveitis	
	Intermediate uveitis	
	Posterior uveitis	
	Endogenous endophthalmitis	
	Ocular microbiology	
	Vitritis	
	Acute retinal necrosis	
	Ocular toxoplasmosis	

PUBMED

MESH

#1 (("high throughput nucleotide sequencing"[MeSH Terms]) OR ("metagenomics"[MeSH Terms])) OR ("molecular diagnostic techniques"[MeSH Terms]) – 87.309

#2 (((((((("uveitis"[MeSH Terms]) OR ("panuveitis"[MeSH Terms])) OR ("iritis"[MeSH Terms])) OR ("chorioretinitis"[MeSH Terms])) OR ("retinitis"[MeSH Terms])) OR ("eye infections"[MeSH Terms])) OR ("pars planitis"[MeSH Terms])) OR ("iridocyclitis"[MeSH Terms])) OR ("panophthalmitis"[MeSH Terms])) OR ("choroiditis"[MeSH Terms]) – 75.873

#1 AND #2

#3 ((("high throughput nucleotide sequencing"[MeSH Terms]) OR ("metagenomics"[MeSH Terms])) OR ("molecular diagnostic techniques"[MeSH Terms])) AND ((((((((("uveitis"[MeSH Terms]) OR ("panuveitis"[MeSH Terms])) OR ("iritis"[MeSH Terms])) OR ("chorioretinitis"[MeSH Terms])) OR ("retinitis"[MeSH Terms])) OR ("eye infections"[MeSH Terms])) OR ("pars planitis"[MeSH Terms])) OR

("iridocyclitis"[MeSH Terms])) OR ("panophthalmitis"[MeSH Terms])) OR ("choroiditis"[MeSH Terms])) – 119

#4 (("genetic diseases, y linked"[MeSH Terms]) OR ("genetic diseases, y linked"[MeSH Terms])) OR ("genetic diseases, inborn"[MeSH Terms])

#3 NOT #4

#5 (((("high throughput nucleotide sequencing"[MeSH Terms]) OR ("metagenomics"[MeSH Terms])) OR ("molecular diagnostic techniques"[MeSH Terms])) AND ((((((((("uveitis"[MeSH Terms]) OR ("panuveitis"[MeSH Terms])) OR ("iritis"[MeSH Terms])) OR ("chorioretinitis"[MeSH Terms])) OR ("retinitis"[MeSH Terms])) OR ("eye infections"[MeSH Terms])) OR ("pars planitis"[MeSH Terms])) OR ("iridocyclitis"[MeSH Terms])) OR ("panophthalmitis"[MeSH Terms])) OR ("choroiditis"[MeSH Terms]))) NOT ((("genetic diseases, y linked"[MeSH Terms]) OR ("genetic diseases, y linked"[MeSH Terms])) OR ("genetic diseases, inborn"[MeSH Terms])) – 106

TITLE/ABSTRACT

#6 ((((((("high throughput nucleotide sequencing"[Title/Abstract]) OR ("metagenomics"[Title/Abstract])) OR ("metagenomic"[Title/Abstract])) OR ("molecular diagnostic techniques"[Title/Abstract])) OR ("molecular diagnostic technique"[Title/Abstract])) OR ("next generation sequencing"[Title/Abstract])) OR ("mngs"[Title/Abstract])) OR ("ngs"[Title/Abstract]) – 98.211

#7 ((((((((((((((((((("uveitis"[Title/Abstract]) OR ("panuveitis"[Title/Abstract])) OR ("iritis"[Title/Abstract])) OR ("chorioretinitis"[Title/Abstract])) OR ("retinitis"[Title/Abstract])) OR ("eye infections"[Title/Abstract])) OR ("eye infection"[Title/Abstract])) OR ("pars planitis"[Title/Abstract])) OR ("iridocyclitis"[Title/Abstract])) OR ("panophthalmitis"[Title/Abstract])) OR ("choroiditis"[Title/Abstract])) OR ("infectious uveitis"[Title/Abstract])) OR ("diffuse uveitis"[Title/Abstract])) OR ("anterior uveitis"[Title/Abstract])) OR ("anterior uveitis"[Title/Abstract])) OR ("intermediate uveitis"[Title/Abstract])) OR ("posterior uveitis"[Title/Abstract])) OR ("endogenous endophthalmitis"[Title/Abstract])) OR ("ocular microbiology"[Title/Abstract])) OR ("vitritis"[Title/Abstract])) OR ("acute retinal necrosis"[Title/Abstract])) OR ("ocular toxoplasmosis"[Title/Abstract]) – 47.970

#6 AND #7

#8 (((((((("high throughput nucleotide sequencing"[Title/Abstract]) OR ("metagenomics"[Title/Abstract])) OR ("metagenomic"[Title/Abstract])) OR ("molecular diagnostic techniques"[Title/Abstract])) OR ("molecular diagnostic technique"[Title/Abstract])) OR ("next generation sequencing"[Title/Abstract])) OR ("mngs"[Title/Abstract])) OR ("ngs"[Title/Abstract])) AND (((((((((((((((((((((("uveitis"[Title/Abstract]) OR ("panuveitis"[Title/Abstract])) OR ("iritis"[Title/Abstract])) OR ("chorioretinitis"[Title/Abstract])) OR ("retinitis"[Title/Abstract])) OR ("eye infections"[Title/Abstract])) OR ("eye infection"[Title/Abstract])) OR ("pars planitis"[Title/Abstract])) OR ("iridocyclitis"[Title/Abstract])) OR ("panophthalmitis"[Title/Abstract])) OR ("choroiditis"[Title/Abstract])) OR ("infectious uveitis"[Title/Abstract])) OR ("diffuse uveitis"[Title/Abstract])) OR ("anterior uveitis"[Title/Abstract])) OR ("anterior uveitis"[Title/Abstract])) OR ("intermediate uveitis"[Title/Abstract])) OR ("posterior uveitis"[Title/Abstract])) OR ("endogenous endophthalmitis"[Title/Abstract])) OR ("ocular

microbiology"[Title/Abstract])) OR ("vitritis"[Title/Abstract])) OR ("acute retinal necrosis"[Title/Abstract])) OR ("ocular toxoplasmosis"[Title/Abstract])) – 461

#9 ((("genetic diseases"[Title/Abstract]) OR ("genetic disease"[Title/Abstract])) OR ("inherited diseases"[Title/Abstract])) OR ("inherited disease"[Title/Abstract]) – 25.001

#8 NOT #9

#10 (((((((("high throughput nucleotide sequencing"[Title/Abstract]) OR ("metagenomics"[Title/Abstract])) OR ("metagenomic"[Title/Abstract])) OR ("molecular diagnostic techniques"[Title/Abstract])) OR ("molecular diagnostic technique"[Title/Abstract])) OR ("next generation sequencing"[Title/Abstract])) OR ("mngs"[Title/Abstract])) OR ("ngs"[Title/Abstract])) AND (((((((((((((((((((("uveitis"[Title/Abstract]) OR ("panuveitis"[Title/Abstract])) OR ("iritis"[Title/Abstract])) OR ("chorioretinitis"[Title/Abstract])) OR ("retinitis"[Title/Abstract])) OR ("eye infections"[Title/Abstract])) OR ("eye infection"[Title/Abstract])) OR ("pars planitis"[Title/Abstract])) OR ("iridocyclitis"[Title/Abstract])) OR ("panophthalmitis"[Title/Abstract])) OR ("choroiditis"[Title/Abstract])) OR ("infectious uveitis"[Title/Abstract])) OR ("diffuse uveitis"[Title/Abstract])) OR ("anterior uveitis"[Title/Abstract])) OR ("anterior uveitis"[Title/Abstract])) OR ("intermediate uveitis"[Title/Abstract])) OR ("posterior uveitis"[Title/Abstract])) OR ("endogenous endophthalmitis"[Title/Abstract])) OR ("ocular microbiology"[Title/Abstract])) OR ("vitritis"[Title/Abstract])) OR ("acute retinal necrosis"[Title/Abstract])) OR ("ocular toxoplasmosis"[Title/Abstract]))) NOT (((("genetic diseases"[Title/Abstract]) OR ("genetic disease"[Title/Abstract])) OR ("inherited diseases"[Title/Abstract])) OR ("inherited disease"[Title/Abstract])) – 445

TEXT WORD

#11 (((((((("high throughput nucleotide sequencing"[Text Word]) OR ("metagenomics"[Text Word])) OR ("metagenomic"[Text Word])) OR ("molecular diagnostic techniques"[Text Word])) OR ("molecular diagnostic technique"[Text Word])) OR ("next generation sequencing"[Text Word])) OR ("mngs"[Text Word])) OR ("ngs"[Text Word])) AND (((((((((((((((((((("uveitis"[Text Word]) OR ("panuveitis"[Text Word])) OR ("iritis"[Text Word])) OR ("chorioretinitis"[Text Word])) OR ("retinitis"[Text Word])) OR ("eye infections"[Text Word])) OR ("eye infection"[Text Word])) OR ("pars planitis"[Text Word])) OR ("iridocyclitis"[Text Word])) OR ("panophthalmitis"[Text Word])) OR ("choroiditis"[Text Word])) OR ("infectious uveitis"[Text Word])) OR ("diffuse uveitis"[Text Word])) OR ("anterior uveitis"[Text Word])) OR ("anterior uveitis"[Text Word])) OR ("intermediate uveitis"[Text Word])) OR ("posterior uveitis"[Text Word])) OR ("endogenous endophthalmitis"[Text Word])) OR ("ocular microbiology"[Text Word])) OR ("vitritis"[Text Word])) OR ("acute retinal necrosis"[Text Word])) OR ("ocular toxoplasmosis"[Text Word]))) NOT (((("genetic diseases"[Text Word]) OR ("genetic disease"[Text Word])) OR ("inherited diseases"[Text Word])) OR ("inherited disease"[Text Word])) - 600

#5 OR #10 OR #11

#12 (((((("high throughput nucleotide sequencing"[MeSH Terms]) OR ("metagenomics"[MeSH Terms])) OR ("molecular diagnostic techniques"[MeSH Terms])) AND ((((((((("uveitis"[MeSH Terms]) OR ("panuveitis"[MeSH Terms])) OR ("iritis"[MeSH Terms])) OR ("chorioretinitis"[MeSH Terms])) OR ("retinitis"[MeSH

Terms])) OR ("eye infections"[MeSH Terms])) OR ("pars planitis"[MeSH Terms])) OR ("iridocyclitis"[MeSH Terms])) OR ("panophthalmitis"[MeSH Terms])) OR ("choroiditis"[MeSH Terms]))) NOT ((("genetic diseases, y linked"[MeSH Terms]) OR ("genetic diseases, y linked"[MeSH Terms])) OR ("genetic diseases, inborn"[MeSH Terms]))) OR ((((((((("high throughput nucleotide sequencing"[Title/Abstract]) OR ("metagenomics"[Title/Abstract])) OR ("metagenomic"[Title/Abstract])) OR ("molecular diagnostic techniques"[Title/Abstract])) OR ("molecular diagnostic technique"[Title/Abstract])) OR ("next generation sequencing"[Title/Abstract])) OR ("mngs"[Title/Abstract])) OR ("ngs"[Title/Abstract])) AND (((((((((((((((((((("uveitis"[Title/Abstract]) OR ("panuveitis"[Title/Abstract])) OR ("iritis"[Title/Abstract])) OR ("chorioretinitis"[Title/Abstract])) OR ("retinitis"[Title/Abstract])) OR ("eye infections"[Title/Abstract])) OR ("eye infection"[Title/Abstract])) OR ("pars planitis"[Title/Abstract])) OR ("iridocyclitis"[Title/Abstract])) OR ("panophthalmitis"[Title/Abstract])) OR ("choroiditis"[Title/Abstract])) OR ("infectious uveitis"[Title/Abstract])) OR ("diffuse uveitis"[Title/Abstract])) OR ("anterior uveitis"[Title/Abstract])) OR ("anterior uveitis"[Title/Abstract])) OR ("intermediate uveitis"[Title/Abstract])) OR ("posterior uveitis"[Title/Abstract])) OR ("endogenous endophthalmitis"[Title/Abstract])) OR ("ocular microbiology"[Title/Abstract])) OR ("vitritis"[Title/Abstract])) OR ("acute retinal necrosis"[Title/Abstract])) OR ("ocular toxoplasmosis"[Title/Abstract]))) NOT (((("genetic diseases"[Title/Abstract]) OR ("genetic disease"[Title/Abstract])) OR ("inherited diseases"[Title/Abstract])) OR ("inherited disease"[Title/Abstract])))) OR ((((((((("high throughput nucleotide sequencing"[Text Word]) OR ("metagenomics"[Text Word])) OR ("metagenomic"[Text Word])) OR ("molecular diagnostic techniques"[Text Word])) OR ("molecular diagnostic technique"[Text Word])) OR ("next generation sequencing"[Text Word])) OR ("mngs"[Text Word])) OR ("ngs"[Text Word])) AND (((((((((((((((((((("uveitis"[Text Word]) OR ("panuveitis"[Text Word])) OR ("iritis"[Text Word])) OR ("chorioretinitis"[Text Word])) OR ("retinitis"[Text Word])) OR ("eye infections"[Text Word])) OR ("eye infection"[Text Word])) OR ("pars planitis"[Text Word])) OR ("iridocyclitis"[Text Word])) OR ("panophthalmitis"[Text Word])) OR ("choroiditis"[Text Word])) OR ("infectious uveitis"[Text Word])) OR ("diffuse uveitis"[Text Word])) OR ("anterior uveitis"[Text Word])) OR ("anterior uveitis"[Text Word])) OR ("intermediate uveitis"[Text Word])) OR ("posterior uveitis"[Text Word])) OR ("endogenous endophthalmitis"[Text Word])) OR ("ocular microbiology"[Text Word])) OR ("vitritis"[Text Word])) OR ("acute retinal necrosis"[Text Word])) OR ("ocular toxoplasmosis"[Text Word]))) NOT (((("genetic diseases"[Text Word]) OR ("genetic disease"[Text Word])) OR ("inherited diseases"[Text Word])) OR ("inherited disease"[Text Word]))) - 650

Supplemental S2: Standardized Data Abstract Form

1) First author last name:____
2) Publication year:____
3) Study location (City, State, Country):___________________________________
4) Reviewer's initials:____

Part 1.

Basic Inclusion/Exclusion Criteria:

1. Did the study evaluate **metagenomic next-generation sequencing (mNGS)**?
   □ Yes □No (if no, exclude it)
2. Did the study evaluate **mNGS vs. other diagnostic methods (culture or conventional microbiological tests (e.g., serologic test, antigenic test, or nucleic acid amplification [PCR] test):___________________________[write here])**? □ Yes □No (*if no, we will not exclude it. We will keep this information to decide in the future. It will depend on the number of studies that we have found*)
3. Did the study evaluate **infectious uveitis (iris, ciliary body, choroid, and endogenous endophthalmitis)?** □Yes □No (if no, exclude it)
4. Duration of study:___________(in weeks or months)
5. Do you believe this study should be excluded? □ Yes □No
6. If yes, why?
   ____

Part 2.

Exposure and Outcomes Assessment (Check more than one if it is necessary)

1. Where did the study take place?
   □ An academic medical center
   □ A community hospital
   □ A nursing homes
   □ Other: ____

2. Which type is this study design?
   □ Retrospective Cohort study
   □ Prospective Cohort study
   □ Case-control study
   □ Randomized controlled trial
   □ Quasi-experimental study
   □ Transversal study

3. Was the study performed in more than one hospital? If yes, please add the number of hospitals
   □Yes (If yes, please add the # of hospitals) ___ □ No

4. Which type of mNGS test? (you can circle more than one)
   □ DNA and RNA testing

   □ DNA testing

   □ RNA testing

   □ Karius test (This is a DNA testing but qualitative/quantitative – you can circle more than one)

   □ Other test (which name:____________________)

   □ Not reported

5. Which type of sequencer was used?
   □ Illumina platform
   □ Oxford Nanopore Sequencers
   □ PacBio (Pacific Biosciences) Sequencers

   □ **Ion Torrent Sequencers**

   □ BGI (Beijing Genomics Institute) or MGI Sequencers
   □ Other:_________________________
   □ Not reported

6. What type is the studied sample? (Check more than one if it is necessary)
   □ aqueous fluid or aqueous humor
   □ vitreous fluid or vitreous humor
   □ Other:____

7. Did the study evaluate **people with immunocompromised conditions**? □ Yes □No

8. How did the study define an **immunocompromised/immunosuppressed** person?

□ Medications: corticosteroids, chemotherapy or other immunosuppressive medications

□ HIV

□ Solid organ transplant □ Hematopoietic stem cell transplant

□ Thalassemia

□ Active cancer (current cancer or in treatment or received diagnosis within last 12 months)

□ Others: ______________________________________

9. **Population characteristics of patients with infectious uveitis** (Fill out only IF the information is available on the papers)

	**Infectious disease**	**Non-Infectious disease**
Total number and %		

Age (Mean [SD] or Median [IQR]):		
% of female		
Length of hospital stay, days		
Type of antibiotics		
Duration of antibiotics treatment, days		

10. **Diagnostic test characteristics of patients with infectious uveitis** (Fill out only IF the information is available on the papers)

	**mNGS**	**culture**	**Other test(s) (____________)**
Total number			
Detection rate of rare pathogens (%)			
Name of the pathogens (bacteria, fungus, virus or mycobacteria). If it is described compared between diagnostic methods			
Coinfection?<br><br>(If yes, type the pathogens)			
Time spent on diagnosis (days)			

What was the earliest test to make the diagnosis?			
Length of hospital stay, days			
Duration of antibiotics treatment, days			
Cost (mean or median)			

11. Was there advantage of mNGS compared to traditional pathogen diagnostic methods for infectious uveitis: □ Yes □No (Describe the conclusions of this advantage or not):____
____
____
____
____
____
____
____
____
____

12. What kind of barriers or challenges did the authors mention when using mNGS or implementing it in the clinical setting?:____
____
____
____
____
____
____
____
____
____
____
____

13. Did the study review the reasoning behind NGS testing? □ Yes □No
If yes, which reasons were given? Please share any quantitative data gathered by the study.

____
____
____
____
____
_____

14. Did the study investigate the clinical impact of mNGS (e.g. whether there was a change in management or treatment of the patient following mNGS results, etc.)? □ Yes □No

Please share the kind of impact and any quantitative data gathered by the study.

For example, a study might show that mNGS had a positive impact in the analysis of 100 cases (10%), such as through new diagnosis in 80 cases (5%).

____
____
____
____
____
______

Part 3. Unadjusted and adjusted associations

1. Raw numbers: Please fill raw data for the following tables if available. Only include measures of effect if they are listed in the manuscript

Table A: Main Association of Interest – mNGS and final clinical diagnosis [reference standard*] (total number)

For the clinical diagnosis or "clinical adjudication", experts can consider as a *reference standard some microbiology methods as a culture, a serology test, antigenic test, etc. Please specify it when filling out the table if possible (all methods or culture or serology test, etc.)

	Clinical diagnosis +	Clinical diagnosis -
Diagnosis from mNGS +		
Diagnosis from mNGS -		

Diagnostic performance:

Sensitivity:

Specificity:

Positive predictive value (PPV):

Negative predictive value (NPV):

Table B: Main Association of Interest – Conventional microbiological test (CMT) [reference standard*] and final clinical diagnosis (total number)

******_**Reference standard can be a culture, a serology test, antigenic test, etc. Please specify it when filling out the table if possible <u>(all methods or culture or serology test, etc.)</u>**_

	Clinical diagnosis +	Clinical diagnosis -
CMT +		
CMT -		

Diagnostic performance:

Sensitivity:

Specificity:

Positive predictive value (PPV):

Negative predictive value (NPV):

Table C: Main Association of Interest – mNGS and CMT [reference standard*] (total number)

******_**Reference standard can be a culture, a serology test, antigenic test, etc. Please specify it when filling out the table if possible <u>(all methods or culture or serology test, etc.)</u>**_

	CMT +	CMT -
Diagnosis from mNGS +		
Diagnosis from mNGS -		

Diagnostic performance:

Sensitivity:

Specificity:

Positive predictive value (PPV):

Negative predictive value (NPV):

Part 4. Other references

Please look through the references. Are there other references that we should evaluate for the meta-analysis? If yes, please provide first author, journal and year

____
____
____
____

Part 5: Quality Assessment Tool:

Adapted Downs and Black Tool:

1. *Is the hypothesis/aim/objective of the study clearly described*?

yes	1
no	0

2. *Are the main outcomes to be measured clearly described in the Introduction or Methods section?*

If the main outcomes are first mentioned in the Results section, the question should be answered no.

yes	1
no	0

3. *Are the characteristics of the participants include in the study clearly described?*

In cohort and cross-sectional studies, inclusion and/or exclusion criteria should be given. In case-control studies, a case-definition and the sources for controls should be given.

yes	1
no	0

4. *Are the interventions of interest clearly described?*

Treatments and placebo (where relevant) that are to be compared should be clearly described.

yes	1
no	0

5. Are the distributions of principal confounders in each group of subjects to be compared clearly described? A list of principal confounders is provided.

yes	2
partially	1
no	0

6. Are the main findings of the study clearly described?

Simple outcome data (including denominators and numerators) should be reported for all major findings so that the reader can check the major analyses and conclusions. (This question does not cover statistical tests which are considered below).

yes	1
no	0

7. Does the study provide estimates of the random variability in the data for the main outcomes?

In non-normally distributed data the inter-quartile range of results should be reported. In normally distributed data the standard error, standard deviation or confidence intervals should be reported. If the distribution of the data is not described, it must be assumed that the estimates used were appropriate and the question should be answered yes.

yes	1
no	0

8. *Have all important adverse events that may be a consequence of the intervention been reported?*

This should be answered yes if the study demonstrates that there was a comprehensive attempt to measure adverse events. (A list of possible adverse events is provided).

yes	1
no	0

9. Have the characteristics of patients lost to follow-up been described?

This should be answered yes where there were no losses to follow-up or where losses to follow-up were so small that findings would be unaffected by their inclusion. This should be answered no where a study does not report the number of patients lost to follow-up.

yes	1
no	0

10. Have actual probability values been reported (e.g. 0.035 rather than <0.05) for the main outcomes except where the probability value is less than 0.001?

yes	1
no	0

External validity:

All the following criteria attempt to address the representativeness of the findings of the study and whether they may be generalized to the population from which the study subjects were derived.

11. Were the subjects asked to participate in the study representative of the entire population from which they were recruited?

The study must identify the source population for patients and describe how the patients were selected. Patients would be representative if they comprised the entire source population, an unselected sample of consecutive patients, or a random sample. Random sampling is only feasible where a list of all members of the relevant population exists. Where a study does not report the proportion of the source population from which the patients are derived, the question should be answered as unable to determine.

yes	1
no	0
unable to determine	0

12. Were those subjects who were prepared to participate representative of the entire population from which they were recruited?

The proportion of those asked who agreed should be stated. Validation that the sample was representative would include demonstrating that the distribution of the main confounding factors was the same in the study sample and the source population.

yes	1
no	0
unable to determine	0

13. *Were the staff, places, and facilities where the patients were treated, representative of the treatment the majority of patients receive?*

For the question to be answered yes the study should demonstrate that the intervention was representative of that in use in the source population. The question should be answered no if, for example, the intervention was undertaken in a specialist center unrepresentative of the hospitals most of the source population would attend.

yes	1
no	0
unable to determine	0

Internal validity – bias

14. *Was an attempt made to blind study subjects to the intervention they have received?*

For studies where the patients would have no way of knowing which intervention they received, this should be answered yes.

yes	1
no	0
unable to determine	0

15. *Was an attempt made to blind those measuring the main outcomes of the intervention?*

yes	1
no	0
unable to determine	0

16. If any of the results of the study were based on "data dredging", was this made clear?

Any analyses that had not been planned at the outset of the study should be clearly indicated. If no retrospective unplanned subgroup analyses were reported, then answer yes.

yes	1
no	0
unable to determine	0

17. In trials and cohort studies, do the analyses adjust for different lengths of follow-up of patients, or in case-control studies, is the time period between the intervention and outcome the same for cases and controls ?

Where follow-up was the same for all study patients the answer should yes. If different lengths of follow-up were adjusted for by, for example, survival analysis the answer should be yes. Studies where differences in follow-up are ignored should be answered no.

yes	1
no	0

unable to determine	0

18. Were the statistical tests used to assess the main outcomes appropriate?
The statistical techniques used must be appropriate to the data. For example non-parametric methods should be used for small sample sizes. Where little statistical analysis has been undertaken but where there is no evidence of bias, the question should be answered yes. If the distribution of the data (normal or not) is not described it must be assumed that the estimates used were appropriate and the question should be answered yes.

yes	1
no	0
unable to determine	0

19. Was compliance with the intervention/s reliable? Where there was non compliance with the allocated treatment or where there was contamination of one group, the question should be answered no. For studies where the effect of any misclassification was likely to bias any association to the null, the question should be answered yes.

yes	1
no	0
unable to determine	0

20. Were the main outcome measures used accurate (valid and reliable)?
For studies where the outcome measures are clearly described, the question should be answered yes. For studies which refer to other work or that demonstrates the outcome measures are accurate, the ques- tion should be answered as yes.

yes	1
no	0
unable to determine	0

Internal validity - confounding (selection bias)

21. Were the patients in different intervention groups (trials and cohort studies) or were the cases and controls (case-control studies) recruited from the same population?
For example, patients for all comparison groups should be selected from the same hospital. The question should be answered unable to determine for cohort and case-

control studies where there is no information concerning the source of patients included in the study.

yes	1
no	0
unable to determine	0

22. Were study subjects in different intervention groups (trials and cohort studies) or were the cases and controls (case-control studies) recruited over the same period of time?
For a study which does not specify the time period over which patients were recruited, the question should be answered as unable to determine.

yes	1
no	0
unable to determine	0

23. *Were study subjects randomised to intervention groups?* Studies which state that subjects were randomised should be answered yes except where method of randomisation would not ensure random allocation. For example alternate allocation would score no because it is predictable.

yes	1
no	0
unable to	0

24. Was the randomised intervention assignment concealed from both patients and health care staff until recruitment was complete and irrevocable?

yes	1
no	0
unable to determine	0

25. Was there adequate adjustment for confounding in the analyses from which the main findings were drawn?
This question should be answered no for trials if: the main conclusions of the study were based on analyses of treatment rather than intention to treat; the distribution of known confounders in the different treatment groups was not described; or the distribution of known confounders differed between the treatment groups but was not taken into account in the analyses. In non-randomized studies if the effect of the main confounders was not investigated or con- founding was demonstrated but no adjustment was made in the final analyses the question should be answered as no.

yes	1
no	0
unable to	0

26. Were losses of patients to follow-up taken into account?

If the numbers of patients lost to follow-up are not reported, the question should be answered as unable to determine. If the proportion lost to follow-up was too small to affect the main findings, the question should be answered yes.

yes	1
no	0
unable to determine	0

Power

27. Did the study perform calculations to determine sufficient power to detect a clinically important difference?

Sample sizes have been calculated to detect a difference of x% and y%.

Yes	1
No	0

Total score: _____
